# Lectin-Fc(IgG) fusion proteins exhibit antifungal activity against the emerging multidrug-resistant pathogen *Candida auris*

**DOI:** 10.1128/iai.00329-25

**Published:** 2025-10-29

**Authors:** Susana Ruiz Mendoza, Leandro Honorato, Deborah Santos Cintra, Marina da Silva Ferreira, Daniel Zamith-Miranda, Joshua D. Nosanchuk, Leonardo Nimrichter, Allan J. Guimarães

**Affiliations:** 1 Laboratório de Bioquímica e Imunologia das Micoses, Departamento de Microbiologia e Parasitologia, Instituto Biomédico, Universidade Federal Fluminense 28110, Niterói, Brazil; 2 Pós-Graduação em Imunologia e Inflamação, Universidade Federal do Rio de Janeiro 28125, Rio de Janeiro, Brazil; 3 Laboratório de Glicobiologia de Eucariotos, Instituto de Microbiologia Professor Paulo de Góes, Universidade Federal do Rio de Janeiro 28125, Rio de Janeiro, Brazil; 4 Programa de Pós-Graduação em Microbiologia e Parasitologia Aplicadas, Instituto Biomédico, Universidade Federal Fluminense 28110, Niterói, Brazil; 5 Department of Medicine (Division of Infectious Diseases) and Microbiology and Immunology, Albert Einstein College of Medicine 2006, Bronx, New York, USA; 6 Rede Micologia RJ – Fundação de Amparo à Pesquisa do Estado do Rio de Janeiro (FAPERJ) 306542, Rio de Janeiro, Brazil; 7 National Institute of Science and Technology (INCT) in Human Pathogenic Fungi, São Paulo, Brazil; University of California Davis, Davis, California, USA

**Keywords:** fusion proteins, Lectin-Fc(IgG), *Candida auris*, passive immunization, protection

## Abstract

*Candida auris* is an emerging fungal pathogen recognized among the Centers for Disease Control and Prevention’s urgent threats and designated a critical priority by the World Health Organization due to its global spread, high mortality rates, potential for pan-drug resistance, and its persistent transmission within healthcare settings. The clinical management of *C. auris* infections is further hindered by the lack of both rapid/specific diagnosis and effective antifungals. These challenges emphasize the urgent need for alternative therapeutic strategies. In this study, we investigated the antifungal, immunomodulatory, and protective effects of engineered Lectin-Fc(IgG) fusion proteins against a fluconazole-resistant *C. auris* strain. Specifically, Dectin-1-Fc(IgG2a), Dectin-1-Fc(IgG2b), and wheat germ agglutinin (WGA)-Fc(IgG2a) demonstrated dose-dependent binding to key fungal cell wall components, β-1,3-glucan and chitin, with Dectin-1-Fc(IgG2b) exhibiting the highest reactivity, followed by Dectin-1-Fc(IgG2a) and WGA-Fc(IgG2a). *In vitro*, all constructs exhibited fungistatic activity and reduced the biofilm biomass and metabolism, with the Dectin-1-Fc(IgG) variants displaying the most potent effects. As opsonins, Lectin-Fc(IgG)s significantly enhanced the macrophage–yeast association and macrophage-mediated killing of *C. auris*. In a systemic murine *C. auris* infection model, a single therapeutic administration of Dectin-1-Fc(IgG2b) or WGA-Fc(IgG2a) conferred 100% protection, while Dectin-1-Fc(IgG2a) achieved >80% protection, with all treated mice manifesting clinical improvement. Quantification of fungal burden at day 7 post-infection revealed at least a ~1 log reduction in colony-forming units in the spleen, kidney, and liver of Lectin-Fc(IgG)-treated animals. Cytokine profiling indicated a Th1-type-skewed immune response in Lectin-Fc(IgG)-treated mice. Collectively, these findings support the antifungal and immunotherapeutic potential of Lectin-Fc(IgG)s against *C. auris*, offering a novel broad-spectrum strategy to overcome current therapeutic limitations.

## INTRODUCTION

The emerging multidrug-resistant yeast *Candida auris* has rapidly become a serious global health threat since its first isolation from the ear canal of a hospitalized patient in Japan in 2009 (1). Over the past decade, it has rapidly spread across more than 40 countries, raising a worldwide concern due to its high transmissibility, environmental persistence, and propensity to cause healthcare-associated outbreaks (2). In 2020, the U.S. Centers for Disease Control and Prevention issued a clinical emergency alert emphasizing the high mortality rates associated with systemic *C. auris* infections, ranging from 46% to 75% (2, 3). These alarming features have drawn considerable attention from the scientific community to investigate the pathogens’ origin, biology, environmental reservoirs, pathogenesis, the onset of clinical manifestations upon infection, epidemiology, and the resistance mechanisms by this pathogen.

As an opportunistic nosocomial pathogen, *C. auris* is frequently isolated from hospital surfaces and the hands of healthcare personnel. Its efficient transmission leads to persistent colonization of multiple patients’ anatomical sites, including the nares, palms, fingertips, axillae, inguinal creases, and toe webs (4). Approximately 25% of colonized individuals develop candidemia within 60 days, with mortality rates ranging from 25% to 70% (2). *C. auris* infections often occur in critically ill intensive care unit (ICU) patients and may manifest as bloodstream infections, urinary tract infections, otitis, or invasive conditions such as skin abscesses, wound infections, osteomyelitis, myocarditis, and meningitis (2, 5). Despite the efforts to restrict *C. auris* colonization and infections in ICUs, a key contributor to its persistence is its ability to form robust and adherent biofilms on medically relevant substrates (6), which confer resistance to common disinfectants and promote prolonged environmental survival (7, 8).

In addition to its resilience, *C. auris* poses substantial diagnostic and therapeutic challenges that contribute to its growing clinical and public health impact. Conventional phenotyping methods frequently misdiagnose *C. auris* as *Candida haemulonii* (9). The gold standard for accurate identification relies on state-of-the-art techniques such as matrix-assisted laser desorption ionization–time of flight mass spectrometry or sequencing of internal transcribed regions or D1/D2 regions of the 28S ribosomal DNA (10, 11). The rising prevalence of nosocomial infections, particularly in severely debilitated patients, coupled with the high frequency of multidrug resistance (MDR) in *C. auris* (2, 12, 13), underscores the critical need for accurate diagnostics and effective therapeutic strategies (14). Together, these limitations significantly contribute to the high clinical and economic burden associated with *C. auris* infections (15).

These ongoing challenges have continuously fueled the research and development of novel antifungal agents for patients’ clinical management and environmental strategies, including synthetic azole derivatives and drug repurposing approaches to circumvent drug toxicity and accelerate antifungal discovery (16). However, resistance to all three major antifungal classes—azoles, polyenes (e.g., amphotericin B), and echinocandins—has been reported for some *C. auris* isolates of pan-resistant isolates and untreatable infections (2). In recognition of these issues, the World Health Organization has designated *C. auris* as a critical fungal pathogen on its Fungal Priority Pathogens List (17). Taken together, these features highlight the pressing need for continuous surveillance, improved diagnostics, and the development of innovative, broad-spectrum antifungal interventions to combat this emerging global threat.

The absence of resistance associated with immunotherapeutic strategies has renewed the interest of the academic community in developing strategies such as vaccines and antibodies and their applications as biopharmaceuticals. However, active immunization through vaccination is often limited in immunocompromised individuals, the most affected population by invasive fungal infections (18, 19). In this context, passive immunizations with antibodies offer a potentially valuable alternative (20). Nevertheless, most of the efforts have focused on the use of species-specific monoclonal antibodies, usually tested *in vitro* and *in vivo* against a few isolates. In spite of this, a more encouraging and optimistic approach would be to target conserved fungal antigens to produce a broader affinity reagent with pan-antifungal capacity (21).

In this study, we investigated the immunotherapeutic potential of Lectin-Fc(IgG) fusion proteins against a multidrug-resistant *C. auris* strain (MMC1). These chimeric Lectin-Fc(IgG)s combine the glycan-binding domains of fungal-targeting lectins with the Fc effector region of murine immunoglobulins (IgGs). Three variants were generated: a wheat germ agglutinin (WGA)-Fc(IgG2a), which selectively targets chitoligomers, and Dectin-1-Fc(IgG2a) and Dectin-1-Fc(IgG2b), which target β-1,3-glucan- both of which are highly conserved fungal cell wall polysaccharides (22, 23). Previous studies have demonstrated that Lectin-Fc(IgG) proteins exhibit direct antifungal activity against *Candida albicans*, *Cryptococcus neoformans*, *Histoplasma capsulatum*, and *Aspergillus fumigatus* (22–24). Additionally, these proteins act as opsonins, enhancing fungal internalization by macrophages and their antifungal activity. Moreover, administrations of Lectin-Fc(IgG) protein had immunomodulatory properties, resulting in fungal clearance and improved mouse survival in *in vivo* models of candidiasis, cryptococcosis, aspergillosis, and histoplasmosis (22, 23).

Our results revealed that Lectin-Fc(IgG) proteins specifically bind to the *C. auris* cell wall, inhibit fungal growth and biofilm formation, and enhance the macrophage-mediated antifungal responses. Moreover, in a murine systemic *C. auris* infection model, Lectin-Fc(IgG) treatment significantly improved fungal clearance and animal survival. Consequently, these findings support the potential of Lectin-Fc(IgG) molecules as a novel class of broad-spectrum antifungal immunobiologicals for the therapy of infections caused by MDR pathogens such as *C. auris*, in addition to other invasive fungal pathogens such as *C. albicans*, *Cryptococcus neoformans*, *Histoplasma capsulatum*, and *Aspergillus fumigatus* previously reported.

## MATERIALS AND METHODS

### Animal use

Female C57BL/6 mice (6–8 weeks old) were obtained from the Laboratory Animal Center (Núcleo para Animais de Laboratório) at the Fluminense Federal University (Niteroi, Rio de Janeiro, Brazil). Mice were housed under specific-pathogen-free facilities with *ad libitum* access to food and water.

### Fungal strain and growth conditions

The fluconazole-resistant MMC1 strain of *C. auris* was generously provided by Professor Joshua D. Nosanchuk (Albert Einstein College of Medicine, New York, USA). *C. auris* yeast cultures were maintained in 10 mL of Sabouraud broth at 37°C for 48 h at 150 rpm with continuous shaking (25). *C. albicans* SC5314 (ATCC MYA-2876) was cultured and prepared according to the standardized growth conditions described (22, 23). Following this, both cultures were transferred to 100 mL of fresh Sabouraud broth and incubated for an additional 24 h. Yeasts were harvested by centrifugation at 1,100 × *g* for 10 min and washed three times by resuspension/centrifugation with phosphate-buffered saline (PBS, 137 mM NaCl, 2.7 mM KCl, 1.5 mM KH_2_PO_4_, 8.1 mM Na_2_HPO_4_, pH 7.4). After washing, yeasts were enumerated using a hemocytometer and used in subsequent experiments.

### Lectin-Fc(IgG) fusion protein expression and purification

Dectin-1-Fc(IgG2a), Dectin-1-Fc(IgG2b), and WGA-Fc(IgG2a) were constructed and expressed in CHO-k1 as previously described (22, 24). Transfected cells were cultured in Ham’s F-12 medium (HyClone; GE Healthcare Life Sciences, Marlborough, MA, USA) supplemented with 1.2 g/L NaHCO_3_, 10% (vol/vol) fetal bovine serum (FBS) (Cultilab, São Paulo, Brazil), 1.5 g/L yeast extract, 1.5 g/L peptone (Kasvi, Paraná, Brazil), 1% (vol/vol) non-essential amino acids, 1% penicillin–streptomycin (Gibco-Life Technologies, Waltham, MA, USA) and 70 µg/mL Zeocin (Thermo Fisher Scientific, Massachusetts, USA). Cells were incubated at 37°C under 5% CO_2_ until reaching confluence, then maintained for a further 48 h. Supernatants were collected by centrifugation (800 *× g* for 10 min), and Lectin-Fc(IgG)s were purified using a HiTrap Protein A HP column according to the manufacturer’s instructions (GE Healthcare Life Sciences, Singapore). Finally, Lectin-Fc(IgG) concentrations and structural integrity were verified using enzyme-linked immunosorbent assay (ELISA) and Western blotting.

### Lectin-Fc(IgG) binding to *C. auris* cell wall

The binding of Lectin-Fc(IgG) proteins to *C. auris* cell wall glycans was assessed by indirect ELISA as described previously (22–24, 26). Briefly, 96-well microplates were coated with 50 µL/well of a 2 × 10^6^ yeast/mL *C. auris* MMC1 yeast suspension in PBS and incubated overnight at 4°C. The plates were washed three times with tris-buffered saline with Tween 20 (TBS-T) (Tris base saline; 10 mM Tris base, 0.15 M NaCl, and 0.01% Tween 20) using a Multiwash ELISA Plate Washer (Molecular Devices) and blocked with 1% bovine serum albumin (BSA) in TBS-T for 1 h at 37°C. After additional washes, Lectin-Fc(IgG)s were added in serial 1:2 dilutions (starting at 25 µg/mL) and incubated for 1 h at 37°C. After additional washing, the plates were incubated with an antimouse IgG alkaline phosphatase-conjugated antibody (Southern Biotech) for 1  h at 37°C. After washing, plates were developed with 1 mg/mL para-nitrophenyl phosphate substrate solution (Sigma-Aldrich) at room temperature (RT) for 30 min, and absorbances were measured at 405 nm using a SpectraMax microplate reader (Molecular Devices). Binding curves were constructed and analyzed as described (22, 27, 28). The maximum binding capacity (Bmax) and apparent dissociation constant ($K_{d}^{app}$) were calculated by non-linear regression fitting of the ELISA titration curves. The ELISA was conducted in three independent experiments, each with two technical replicates per condition.

Binding of Lectin-Fc(IgG)s to the cell wall of *C. auris* was further evaluated by immunofluorescence and flow cytometry as described previously (22–24). First, 2 × 10^6^ yeasts of *C. auris* yeast MMC1 (previously fixed in 4% paraformaldehyde) were blocked with BSA 1% in PBS for 1 h at RT. Yeasts were washed three times with PB and incubated separately with 5 µg/mL of Lectin-Fc(IgG)s [Dectin-1-Fc(IgG2a), Dectin-1-Fc(IgG2b), or WGA-Fc(IgG2a)] for 1 h at RT in a tube revolver rotator (Thermo Fisher Scientific). After washing, the yeasts were incubated with 1 µg/mL of a goat antimouse IgG-Alexa Fluor 546 (Molecular Probes–Life Technologies) for 1 h at RT, followed by staining with 5 mg/mL Uvitex 2B in PBS for 30 min at RT. Samples were washed and transferred to microscope slides with sterile coverslips for visualization using an Elysa Super-Resolution Microscope (Carl-Zeiss, Inc.) with a ×100 objective. Images were analyzed and processed using Image J (National Institutes of Health, Bethesda, MD, USA) and Adobe Photoshop (Adobe Systems Software). For flow cytometry, samples were analyzed on a FACSCalibur (BD Biosciences, Franklin Lakes, NJ, USA), and fluorescence intensity (FL2) and percentage of fluorescent cells were determined using FlowJo X software (Becton Dickinson, Franklin Lakes, NJ, USA). Unstained (absence of fluorescence) controls included a sample without incubations with Lectin-Fc(IgG)s nor fluorescence conjugate incubations (PBS), whereas a second control consisted of absence of Lectin-Fc(IgG)s and incubations with fluorescence conjugate only. The experiment was performed three times, each with three technical replicates per condition.

### Effects of Lectin-Fc(IgG) proteins on fungal growth

The antifungal assay of Lectin-Fc(IgG)s was previously standardized by our group against *C. albicans*, *H. capsulatum*, *A. fumigatus*, and *C. neoformans* yeast according to Clinical and Laboratory Standards Institute (CLSI) protocols (22–24). *C. auris* growth inhibition assays were performed according to the CLSI protocols with slight modifications (23, 25). In 96-well honeycomb plates, 200 µL of *C. auris MMC1* suspension (1 × 10^3^ yeasts/mL) in RPMI 1640 medium supplemented with 2% glucose was incubated with Lectin-Fc(IgG)s at concentrations ranging from 12.5 to 0.09 µg/mL. Amphotericin B was used as an antifungal control (0.125–16.0 μg/mL). Cultures were incubated at 37°C for approximately 4 days in a Bioscreen (Oy Growth Curves Ab Ltd), and absorbances at 600 nm were recorded hourly. Growth curves were plotted over time, and the cumulative growth was assessed by calculating the area under the curve (AUC), and the Lectin-Fc(IgG)s were compared with untreated controls. The experiment was performed three times, each with three technical replicates per condition.

### Interference of Lectin-Fc(IgG)s on biofilm formation

To evaluate the effect of Lectin-Fc(IgG) proteins on fungal biofilm formation, 2 × 10^6^ MMC1 *C. auris* yeasts/mL were cultured in 100 µL/well of a 96-well plate with RPMI-1640 medium supplemented with 5% FBS (Cultilab) and 1% penicillin–streptomycin (Gibco-Life Technologies) containing a final concentration of 10, 5, and 1 µg/mL of each Lectin-Fc(IgG)s, for 24 and 48 h at 37°C and 5% CO_2_. As positive and negative controls for biofilm formation, PBS or 4 µg/mL of amphotericin B were used, respectively. The biomass was assessed using 1% crystal violet (CV) (Merck, Kenilworth, NJ, USA) for 20 min at RT, as described by Rodriguez-de la Noval (24). The plates were washed and incubated for 5 min with absolute ethanol. After incubation, the cells were transferred to a new plate, and the absorbances were measured at 570 nm using a SpectraMax microplate reader. The metabolic activity of biofilms under the same setups was assessed by the 2,3-bis(2-methoxy-4-nitro-5-sulfophenyl)-5-[(phenylamino)carbonyl]-2H-tetrazolium hydroxide (XTT) assay as described (26). The experiment was performed three times, each with three technical replicates per condition.

### Influence of Lectin-Fc(IgG) proteins on fungal interaction with macrophages

The interaction assay was performed as described (22, 24, 26, 29). Approximately 8 × 10^5^ bone marrow-derived macrophages/well were cultured in 12-well plates overnight in RPMI-1640 medium supplemented with 5% FBS (Cultilab) and 1% penicillin–streptomycin (Gibco-Life Technologies) at 37°C and 5% CO_2_. Yeasts (*C. auris* and *C. albicans*) were washed three times with PBS and labeled with NHS-Rhodamine (Thermo Fisher Scientific) at 40 µg/mL for 1 h at RT, protected from light, and then washed five times with PBS. Yeasts were incubated with either Lectin-Fc(IgG) fusion proteins (5 µg/mL) or PBS as a negative control for 1 h at RT. Before the infection, macrophages were washed with plain RPMI 1640 medium and infected at a 2:1 multiplicity of infection (MOI, yeasts:macrophage) and incubated for 2 h. Non-interacting yeasts were removed by washing three times with PBS. Macrophages were gently detached from the wells by pipetting up and down, and the suspensions were fixed with 4% paraformaldehyde for 20 min at RT. Cells were washed, and interaction rates were assessed in a FACSCalibur. The percentage of fungi-interacting macrophages (%FL2+) was determined using FlowJo X software. The experiment was performed three times, each with two technical replicates per condition.

### Influence of Lectin-Fc(IgG) proteins on macrophage fungicidal activity

To assess the influence of Lectin-Fc(IgG) proteins on fungal growth within macrophages, a colony-forming unit (CFU) inhibition assay was performed (24). Bone marrow-derived macrophages (1 × 10^5^/well) were cultured in 96-well plates for 24 h at 37°C and 5% CO_2_ in Dulbecco’s modified Eagle medium (DMEM) supplemented with 5% FBS and 1% of penicillin–streptomycin. *C. auris* and *C. albicans* yeasts were washed and incubated for 1 h at RT with either 5 µg/mL of either Lectin-Fc(IgG) or PBS (control). Yeast cells were added to macrophages at a 2:1 MOI for 2 h at 37°C in 5% CO_2_. Non-interacting yeasts were removed by washing, and the macrophages were further incubated overnight in fresh DMEM at 37°C and 5% CO_2_. Macrophages were lysed with sterile distilled water, and recovered yeast were plated on Sabouraud agar plates. Plates were incubated for 24 h at 37°C, and CFUs were counted to compare fungal survival across all experimental conditions. The experiment was performed twice, each with three biological replicates and two technical replicates per condition.

### Protective effects of Lectin-Fc(IgG) proteins in an *in vivo* model of *C. auris*

The *in vivo* protective efficacy of Lectin-Fc(IgG) proteins was initially evaluated using previously standardized protocols for the murine disseminated candidiasis model by assessing survival rates and weight recovery following infection (30, 31). BALB/c female mice (6–8 weeks old, *n* = 10 per group) were immunosuppressed with cyclophosphamide (150 mg/kg) and cortisone acetate (112 mg/kg) before intravenous inoculation by tail vein injection with 5 × 10⁵ *C. auris* MMC1 yeasts (in 100 µL PBS). Two hours post-infection, the mice received intraperitoneal injections of 10 µg of each ectin-Fc(IgG)s in 500 µL of PBS or PBS alone (control). Animals were closely monitored daily for clinical signs, such as tachypnea, lethargy, decreased reflexes, and weight changes. Each sign was attributed a score from 0 (normal) to 3 (sick) daily. Animals reaching a total score of 10–12 were considered to be experiencing severe distress and were euthanized to prevent unnecessary suffering. Daily survival rates were recorded.

Moreover, infected and treated mice as described above were euthanized 7 days post-infection, and the lungs, spleens, and kidneys were collected and weighed. Organs were homogenized in PBS (70 µm cell strainer, BD Biosciences) and plated in triplicate onto Sabouraud agar plates for CFU enumeration after 48 h at 37°C. The experiment was performed twice, each with *n* = 5 mice per group.

### Cytokine measurements in organ homogenates

Lung, spleen, and kidney were homogenized in the presence of protease inhibitor cocktail (Complete Mini; Boehringer Ingelheim, Connecticut, USA) and centrifuged at 6,000 × *g* for 10 min for debris removal. Collected supernatants were stored at −80°C until specific cytokine assessment. Supernatants were measured for interferon gamma (IFN-γ), interleukin (IL)-4, and IL-17 by capture ELISA following the manufacturer’s instructions (BD Biosciences). ELISA was performed twice, each with three technical replicates per condition.

### Statistical analysis

All statistical analyes were performed using GraphPad Prism version 9.00 (GraphPad Software, San Diego, CA, USA). One-way analysis of variance was conducted to compare differences among groups, with a 95% confidence interval and *P* ≤ 0.05 considered statistically significant for all experiments. Tukey’s (pairwise) or Dunnett’s (comparisons against the control group) post hoc test was used as appropriate. Survival data were analyzed by Kaplan–Meier survival curves and log-rank estimator, with differences of *P* ≤ 0.05 considered statistically significant.

## RESULTS

### Lectin-Fc(IgG) proteins bind to *C. auris* cell wall components

**Fig 1:**
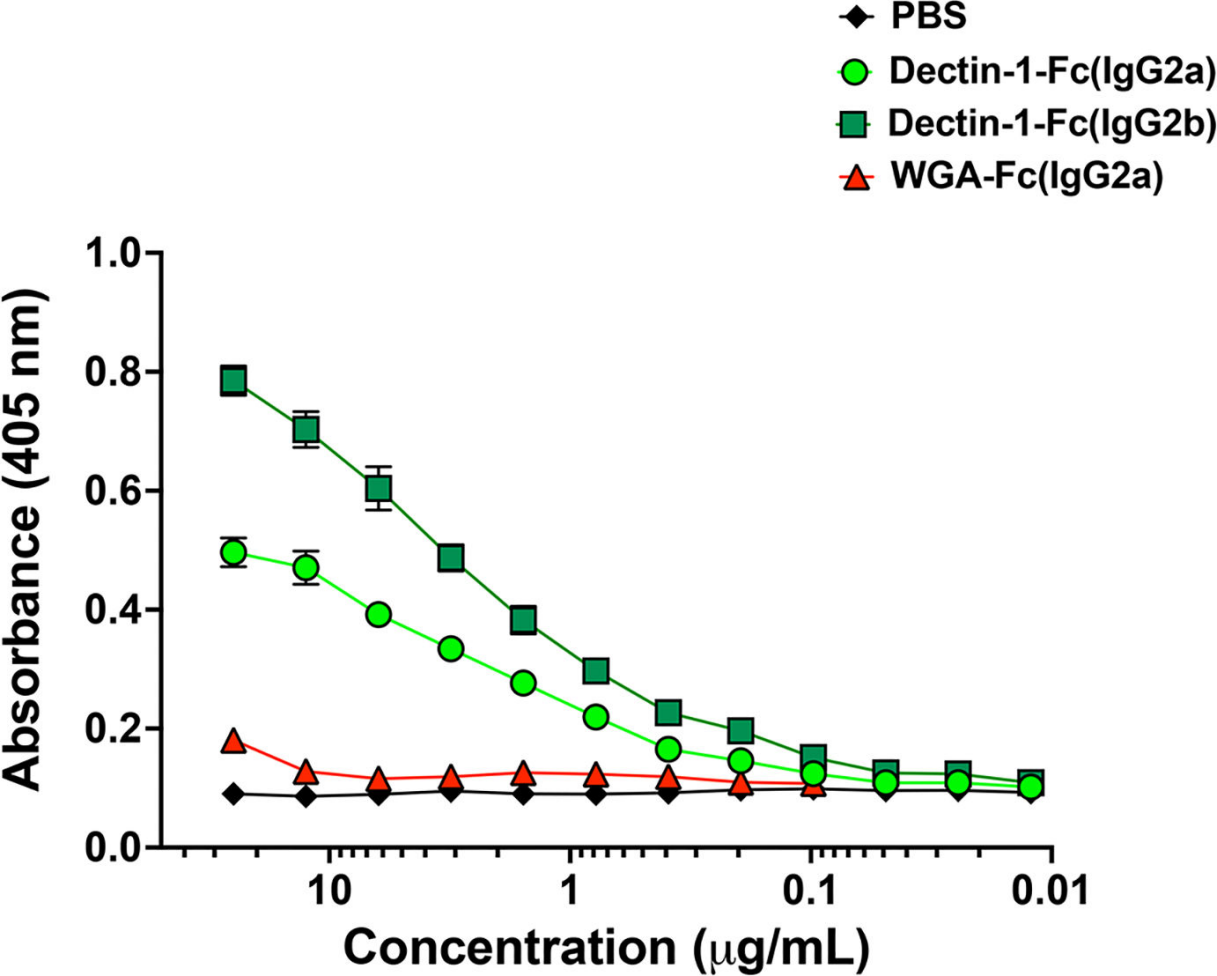
Binding of Lectin-Fc(IgG) fusion proteins to the fluconazole-resistant MMC1 strain of *Candida auris*. Indirect ELISA was performed to evaluate the binding of Dectin-1-Fc(IgG2b) and WGA-Fc(IgG2a) to *C. auris* MMC1 yeasts. Lectin-Fc(IgG)s were added in serial dilutions (0.01–25.0 µg/mL), and binding curves were generated to determine the apparent dissociation constant ($K_{d}^{app}$) and maximum binding capacity (Bmax).

ELISA assays demonstrated that Dectin-1-Fc(IgG)s and WGA-Fc(IgG2a) bind to *C. auris* yeasts in a concentration-dependent manner, reflecting specific recognition of β-1,3-glucan and chitin, respectively. Dectin-1-Fc(IgG2b) displayed the highest binding capacity (Bmax = 0.746 and $K_{d}^{app}$= 8.4 × 10^−9^ mol/L, Fig. 1), followed by Dectin-1-Fc(IgG2a) (Bmax = 0.459 and $K_{d}^{app}$= 6.8 × 10^−9^ mol/L). In contrast, WGA-Fc(IgG2a) exhibited only a discrete binding (Bmax = 0.133 and $K_{d}^{app}$= 3.8 × 10^−11^ mol/L) (Fig. 1), suggesting lower chitin levels or reduced accessibility of this polysaccharide at the *C. auris* surface.

### Lectin-Fc(IgG)s display higher affinity to *C. albicans* than *C. auris* yeasts

**Fig 2:**
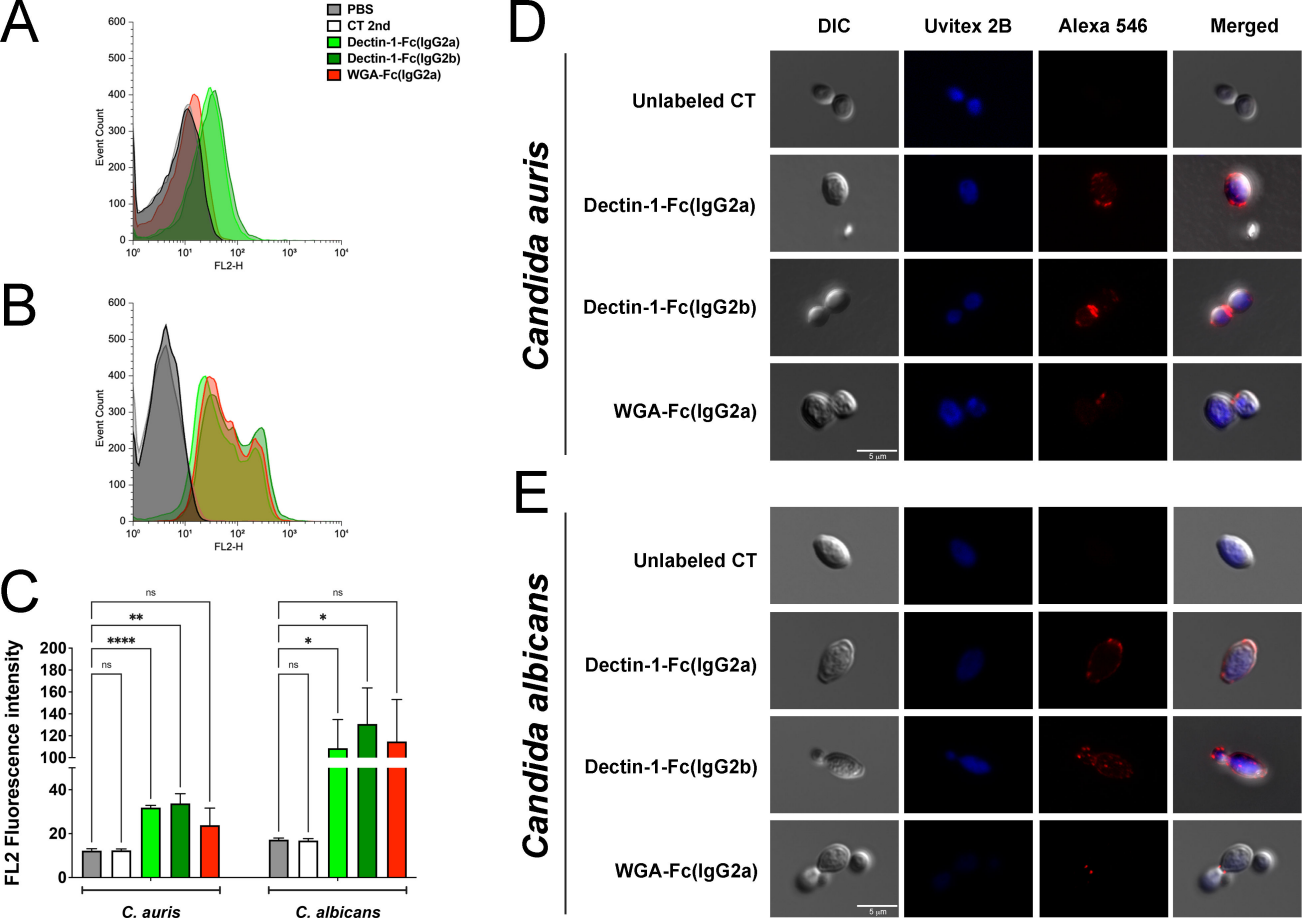
Analysis of Lectin-Fc(IgG)s binding to *C. auris* cell wall components by flow cytometry and immunofluorescence assays. *C. auris* MMC1 and *C. albicans* SC5314 yeasts were incubated with the Lectin-Fc(IgG)s, followed by an antimouse IgG Alexa546-conjugated secondary antibody. Controls included a PBS group [without Lectin-Fc(IgG)s and secondary antibody], and second control (CT 2nd) [yeasts incubated with secondary antibody only, in the absence of Lectin-Fc(IgG)s]. Fluorescence intensities were assessed by flow cytometry analysis and surface binding patterns evaluated by immunofluorescence microscopy imaging. Fluorescence intensity histograms for (**A**) *C. auris* MMC1 and (**B**) *C. albicans* SC5314 and (**C**) mean fluorescence intensity plots show significantly higher binding of Lectin-Fc(IgG) proteins, particularly Dectin-1-Fc(IgG2b) to *C. auris*, in comparison to *C. albicans*. Immunofluorescence microscopy images showing the surface and binding patterns of Lectin-Fc(IgG) fusion proteins to β-1,3-glucan and chitin in (**D**) *C. auris* MMC1 and (**E**) *C. albicans* SC5314 cell wall. Dectin-1-Fc(IgG)s proteins display a punctate annular pattern around the *C. auris* yeast cell wall, while WGA-Fc(IgG2a) is labeled as discrete dots. In contrast, *C. albicans* shows a dotted pattern concentrated around the budding scars. All proteins showed stronger binding to *C. albicans*. Symbols: ns= not significant, *P < 0.05, **P < 0.01, and ****P < 0.0001. DIC, differential interference contrast.

Flow cytometry and immunofluorescence assays were conducted to quantitatively and qualitatively assess and compare the binding profiles of Lectin-Fc(IgG)s to their respective fungal cell wall targets in *C. auris* and *C. albicans*. Consistent with the ELISA data, Dectin-1-Fc(IgG2b) revealed the highest fluorescence intensity (*P* = 0.0071) upon binding to *C. auris*, followed by Dectin-1-Fc(IgG2a) (*P* < 0.0001), while WGA-Fc(IgG2a) displayed little signal (*P* = 0.20, Fig. 2A and C). Binding to *C. albicans* followed a similar trend, although the overall binding of Lectin-Fc(IgG)s and fluorescence intensities were higher compared to *C. auris*, indicating greater target accessibility in *C. albicans* (Fig. 2B and C). Immunofluorescence microscopy confirmed these findings, revealing that Dectin-1-Fc(IgG)s labeling, besides the punctate labeling dispersion across the *C. auris* cell, was concentrated more at the budding regions (Fig. 2D), while in *C. albicans*, Dectin-1-Fc(IgG)s labeling was more dispersed (Fig. 2E). For both yeasts, WGA-Fc(IgG2a) labeling concentrated as small dots at the budding regions, but with lower intensity in *C. auris* than *C. albicans*, aligning with the flow cytometry results.

### Lectin-Fc(IgG) proteins directly inhibit *C. auris* fungal growth

**Fig 3:**
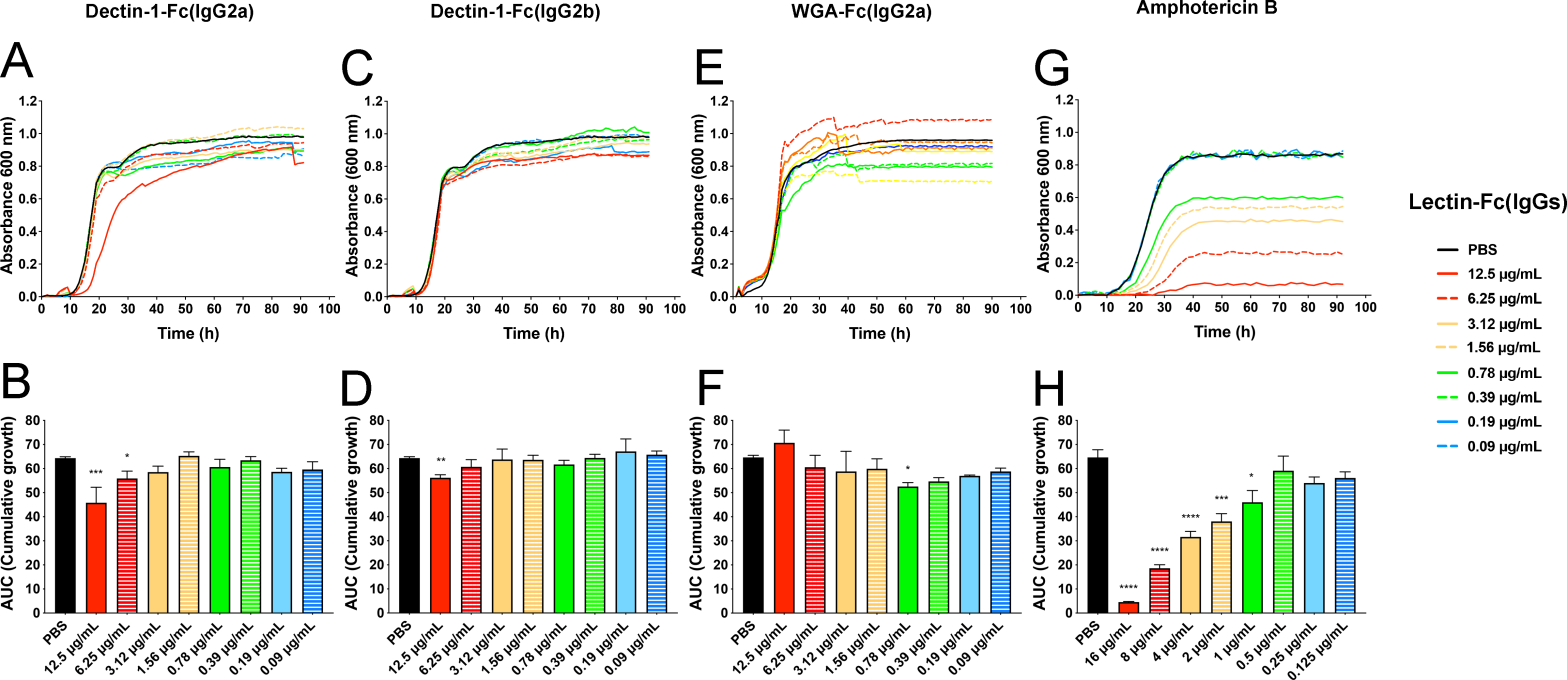
Lectin-Fc(IgG) fusion proteins affect *C. auris* growth *in vitro*. Graphical representation of growth kinetics (**A, C, E, and G**; upper row panels) of *C. auris* co-incubated with varying concentrations (0.097–12.5 µg/mL) of (**A**) Dectin-1-Fc(IgG2a), (**C**) Dectin-1-Fc(IgG2b), (**E**) WGA-Fc(IgG2a), or amphotericin B as a control and assessed by absorbance at 600 nm. (**B, D, F, and H**; lower row panels) Quantification of fungal growth by the area under the curve (AUC), which represents the integral of the growth curves (right panels), demonstrated a dose-dependent fungistatic effect. Symbols: *P < 0.05, **P < 0.0, ***P < 0.001, and ****P < 0.0001.

To assess the direct antifungal potential of Lectin-Fc(IgG)s against *C. auris*, yeasts were cultured in the presence of serial dilutions of Dectin-1-Fc(IgG2a), Dectin-1-Fc(IgG2b), or WGA-Fc(IgG2a). Growth curves and AUC analyses revealed that the Lectin-Fc(IgG)s exerted a dose-dependent fungistatic effect against *C. auris* (Fig. 3A through F). Dectin-1-Fc(IgG2a) at 12.5 µg/mL significantly suppressed fungal growth (*P* = 0.0001), reducing both the exponential growth rate and cumulative biomass production, with statistically inhibitory effects also observed down to 6.25 µg/mL (*P* = 0.037) (Fig. 3A and B). Dectin-1-Fc(IgG2b) displayed significant inhibition only at 12.5 µg/mL (*P* = 0.029) (Fig. 3C and D). Surprisingly, WGA-Fc(IgG2a) inhibited growth significantly only at 0.78 µg/mL (*P* = 0.032, Fig. 3E and F), suggesting that lower protein concentrations may preferentially allow the accessibility of WGA-Fc(IgG2a) to interact with cell wall regions where chitin is more exposed. Collectively, these results indicate that Lectin-Fc(IgG) proteins exert a dose-dependent fungistatic effect on *C. auris*, significantly inhibiting yeast proliferation. Amphotericin B was used as a control for *C. auris* growth inhibition (Fig. 3G and H).

### Lectin-Fc(IgG) proteins reduced *C. auris* biofilm formation and metabolic activity

**Fig 4:**
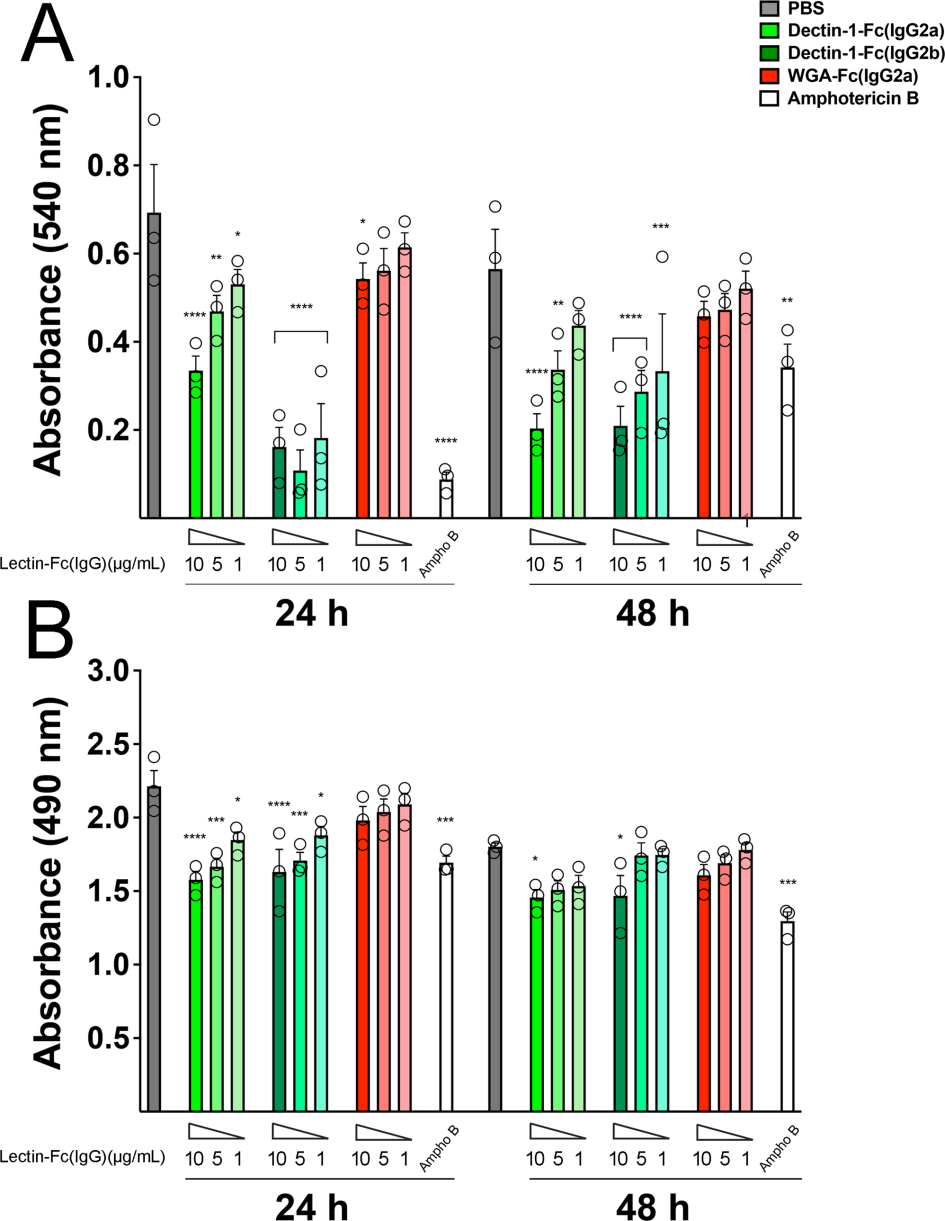
Lectin-Fc(IgG) proteins modulate *C. auris* biofilm formation and metabolic activity. Biofilm formation was evaluated in the presence of Lectin-Fc(IgG) proteins at 10, 5, and 1 µg/mL, PBS (control), and amphotericin B. (**A**) Biofilm biomass was assessed by crystal violet staining at 24 and 48 h incubation following treatment with Dectin-1-Fc(IgG2a), Dectin-1-Fc(IgG2b), or WGA-Fc(IgG2a). (**B**) Metabolic activity of *C. auris* biofilms was determined by the XTT reduction assay. Dectin-1-Fc(IgG) significantly reduced biomass and metabolic activity in a dose-dependent manner, with Dectin-1-Fc(IgG2b) displaying the highest inhibitory efficacy, comparable to amphotericin B. Symbols: *P < 0.05, **P < 0.01, ***P < 0.001, and ****P < 0.0001.

Lectin-Fc(IgG)s altered the biofilm biomass and metabolic activity, as assessed using CV and XTT reduction assays, respectively (Fig. 4A and B). Dectin-1-Fc(IgG2b) significantly inhibited the biofilm biomass development at the three concentrations tested (10, 5, and 1 µg/mL; *P* < 0.0001) after 24 h, comparable to the amphotericin B treatment (*P* = 0.68, *P* = 0.97, and *P* = 0.48, respectively; Fig. 4A). Dectin-1-Fc(IgG2a) also exhibited a dose-dependent inhibitory effect after 24 h, though slightly less pronounced than the Dectin-1-Fc(IgG2b) counterpart. Both Dectin-1-Fc(IgG)s displayed similar inhibitory effects at 10 and 5 µg/mL after 48 h (*P* < 0.01), suggesting a long-lasting antibiofilm activity for both. In turn, WGA-Fc(IgG2a) displayed a modest inhibitory effect at 10 µg/mL at 24 h (*P* = 0.048), reflecting its lower binding affinity to *C. auris*. XTT assays revealed that both Dectin-1-Fc(IgG2a) and Dectin-1-Fc(IgG2b) significantly reduced metabolic activity in a dose-dependent manner at 24 h compared to untreated controls. However, at 48 h, only the highest concentration (10 µg/mL) of both Dectin-1-Fc(IgG2a) and Dectin-1-Fc(IgG2b) maintained a significant reduction in the metabolic activity (*P* = 0.031 and *P* = 0.040, respectively).

### Lectin-Fc(IgG) proteins act as opsonins, enhancing yeast–macrophage interaction and antifungal activity

**Fig 5:**
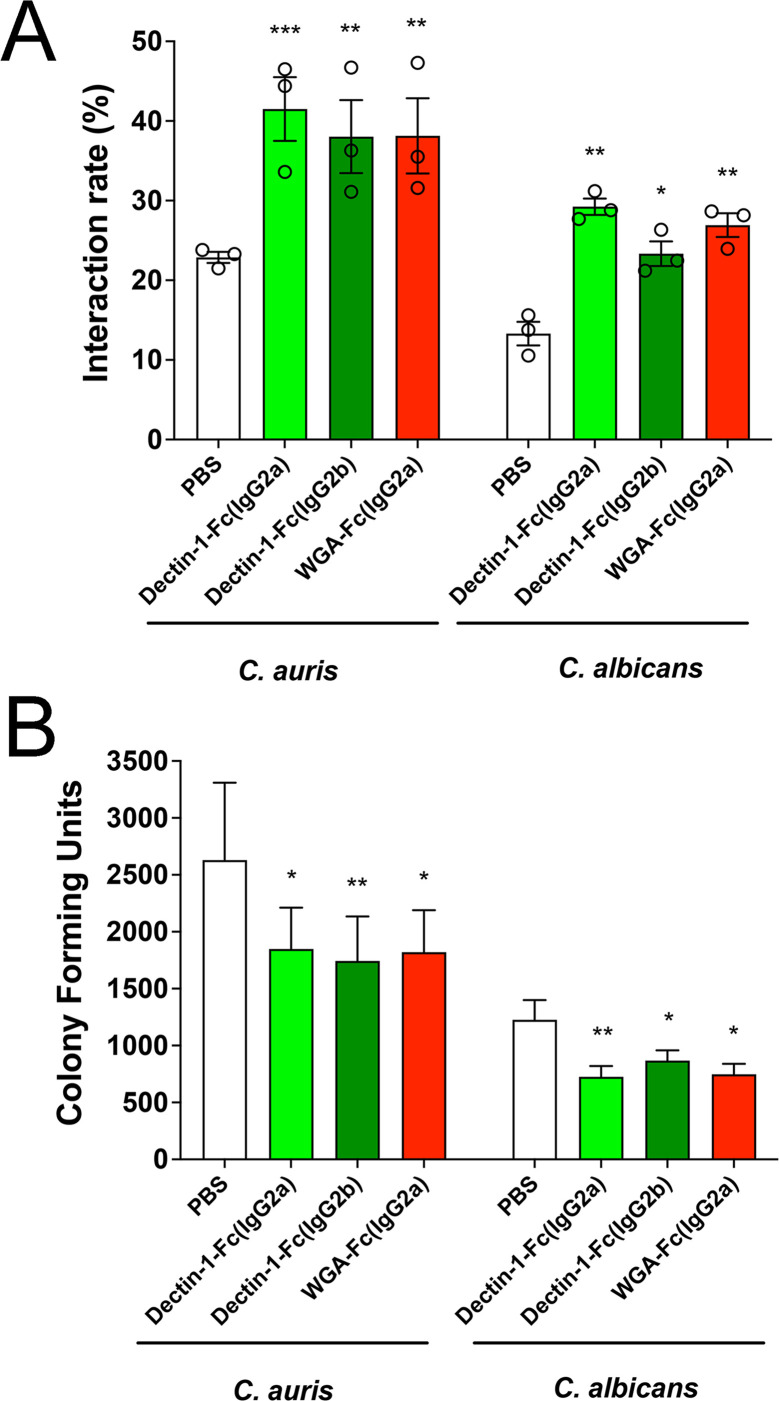
Lectin-Fc (IgG) proteins enhance *C. auris*-macrophage interactions and mediate antifungal activity. *C. auris* and *C. albicans* yeasts were treated with 5 µg/mL of Dectin-1-Fc(IgG2a) (light green), Dectin-1-Fc(IgG2b) (dark green), WGA-Fc(IgG2a) (red), or control (PBS, white). (**A**) The interaction rate of *C. auris* MMC1 and *C. albicans* SC5314 yeasts with macrophages was determined by the ratio of infected macrophages (containing attached or internalized yeasts) to the total number of total macrophages. All proteins significantly increased yeast–macrophage association, with Dectin-1-Fc(IgG2a) showing the highest enhancement in *C. auris*. (**B**) Fungal viability within the macrophage intraphagosomal environment was assessed by CFU quantification of yeasts. All treated groups showed reduced fungal survival in all Lectin-Fc(IgG) treatments, with Dectin-1-Fc(IgG2b) displaying the greatest reduction in *C. auris*. Symbols: *P < 0.05, **P < 0.01, ***P < 0.001, and ****P < 0.0001.

To initially investigate the immunomodulatory potential of Lectin-Fc(IgG) fusion proteins, we evaluated their effects on the yeast–macrophage interaction rates and how they impacted the yeast survival within macrophages upon interaction. Pre-incubation of *C. auris* and *C. albicans* yeasts with Lectin-Fc(IgG)s significantly increased their interaction with macrophages compared to PBS-treated controls (Fig. 5A). For *C. auris*, Dectin-1-Fc(IgG2a) displayed the strongest opsonization (41.5%, *P* = 0.0003), followed closely by WGA-Fc(IgG2a) (38.1%) and Dectin-1-Fc(IgG2b) (38.0%), which also displayed similarly robust opsonization (*P* = 0.0016, Fig. 5A). Similar trends were observed for *C. albicans* used as a control, with Dectin-1-Fc(IgG2a) (29.2%, *P* = 0.013) significantly enhancing the interactions, followed by WGA-Fc(IgG2a) (26.9%, *P* = 0.0044) and Dectin-1-Fc(IgG2b) (23.3%, *P* = 0.027). Overall, the opsonization activity was more pronounced in *C. auris* compared to *C. albicans*. Additionally, opsonization significantly enhanced macrophage-mediated antifungal activity, reducing fungal survival, assessed by determining fungal viability by CFU counting (Fig. 5B). For *C. auris*, Dectin-1-Fc(IgG2b) led to the most substantial reduction in CFUs (*P* = 0.034), followed by Dectin-1-Fc(IgG2a) and WGA-Fc(IgG2a) (*P* = 0.046 and *P* = 0.049, respectively) when compared to PBS-treated yeasts (Fig. 5B). As a control, for *C. albicans*, macrophage antifungal activity was further enhanced compared to *C. auris*, with the most pronounced macrophage-mediated antifungal activity observed for Dectin-1-Fc(IgG2a) (*P* = 0.017), followed by WGA-Fc(IgG2a) (*P* = 0.016) and Dectin-1-Fc(IgG2b) (*P* = 0.025) in comparison to PBS controls (Fig. 5B). These results demonstrate that Lectin-Fc(IgG) proteins act as effective opsonins, significantly enhancing macrophage fungal recognition and killing of *C. auris*.

### Lectin-Fc(IgG) treatment improves mice recovery after *C. auris* infection

**Fig 6:**
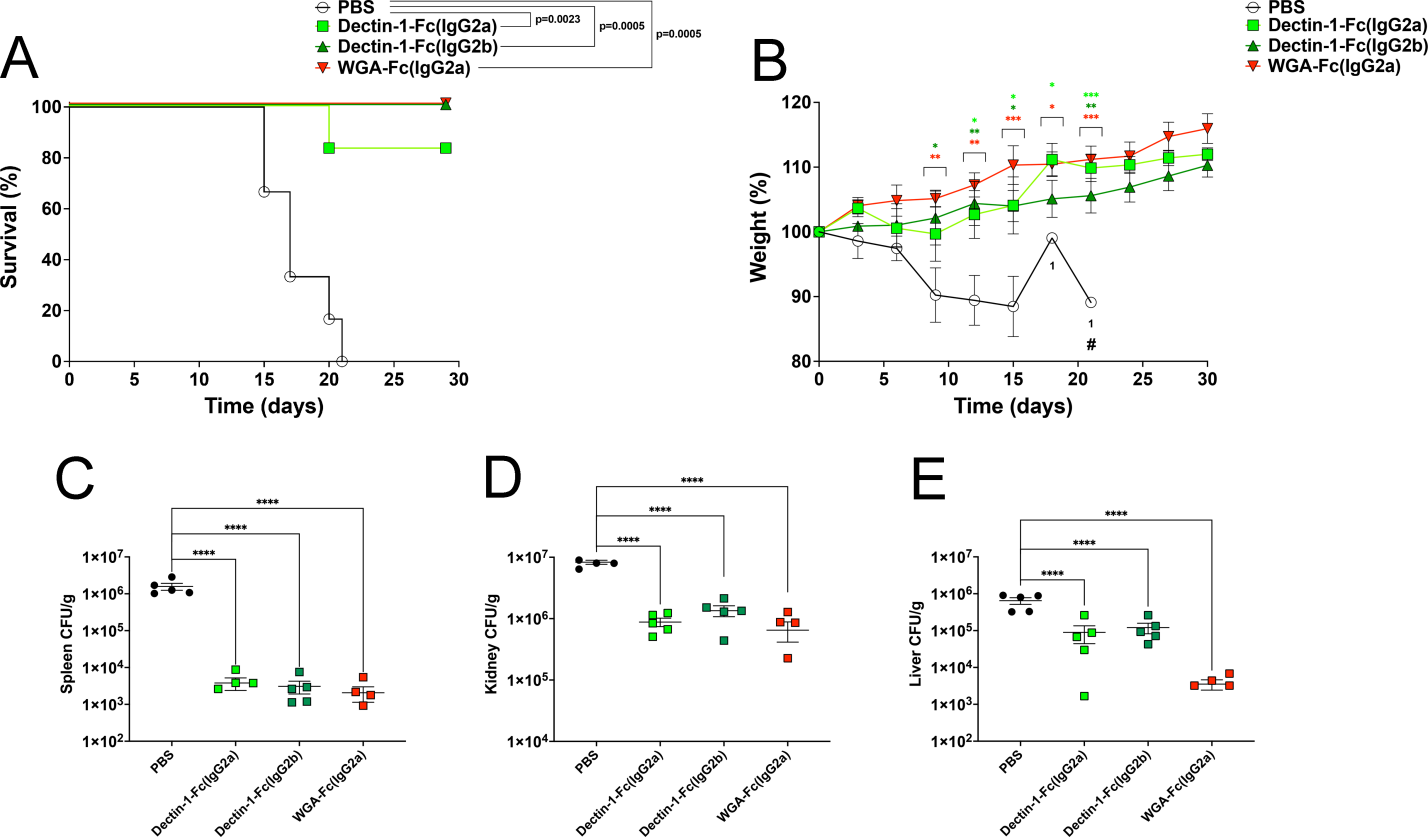
Lectin-Fc(IgG) proteins enhance survival, promote recovery, and reduce fungal burden in a murine model of systemic *Candida auris* infection. The administration of Lectin-Fc(IgG)s was protective in a murine *C. auris* infection model. (**A**) Kaplan–Meier survival curves of *C. auris* infected mice treated with Lectin-Fc(IgG) proteins. Dectin-1-Fc(IgG2b) and WGA-Fc(IgG2a) conferred 100% survival, while Dectin-1-Fc(IgG2a) protected over 80% of mice. All control mice succumbed by day 21 (*P* < 0.01 for all treatments). (**B**) Body weight monitoring in mice after *C .auris* infection revealing weight recovery and clinical improvement in all Lectin-Fc(IgG)-treated groups. (**C–E**) Fungal burden was assessed by CFU quantification in infected organs at 7 days post-infection. (**C**) Spleen, (**D**) kidney, and (**E**) liver. All Lectin-Fc(IgG)s significantly reduced fungal burden in spleen and kidneys (~1 log reduction). Notably, WGA-Fc(IgG2a) showed a 2 log reduction in liver fungal load, performing better than the Dectin-1-Fc(IgG) variants in this organ. Symbols: *P < 0.05, **P < 0.01, ***P < 0.001, and ****P < 0.0001.

Due to the capacity of Lectin-Fc(IgG)s to inhibit *C. auris* growth and to function as opsonins enhancing the antifungal functions of macrophages, their protective efficacy *in vivo* was evaluated. The administration of Lectin-Fc(IgG) fusion proteins conferred significant protection in a systemic murine model of *C. auris* infection, as evidenced by both survival rates and clinical recovery (Fig. 6A and B). Following infection, Dectin-1-Fc(IgG2b) and WGA-F(IgG2a) fully protected the animals (*P* = 0.0005 for both), while Dectin-1-Fc(IgG2a) treatment protected >80% of the mice by day 30 post-infection (*P* = 0.0023). In contrast, all untreated control mice succumbed to infection by day 21 (Fig. 6A). In parallel, as the weight loss is a widely observed manifestation of systemic candidiasis, we followed the body weight of the animals during the course of infection. Within the first week post-infection, mice weight did not demonstrate any difference among groups. However, infected mice treated with Lectin-Fc(IgG)s exhibited marked weight increase and effective resolution of infection, whereas untreated controls experienced progressive clinical deterioration associated with weight loss, culminating in death by day 21 (Fig. 6B).

Quantitative assessment of organ fungal burden at day 7 post-infection revealed that the administration of Lectin-Fc(IgG)s significantly reduced organ colonization and dissemination (Fig. 6C through E). In both the spleen and kidneys, the Lectin-Fc(IgG) treatments induced a significant reduction of about 1 log in organ CFUs (*P* < 0.0001, Fig. 6C and D). Liver fungal loads were similarly reduced by Dectin-1-Fc(IgG2a) and Dectin-1-Fc(IgG2b), while WGA-Fc(IgG2a) elicited a more substantial ~2 log reduction (Fig. 6E).

### Lectin-Fc(IgG) administration modulates cytokine production during *C. auris* infection

**Fig 7:**
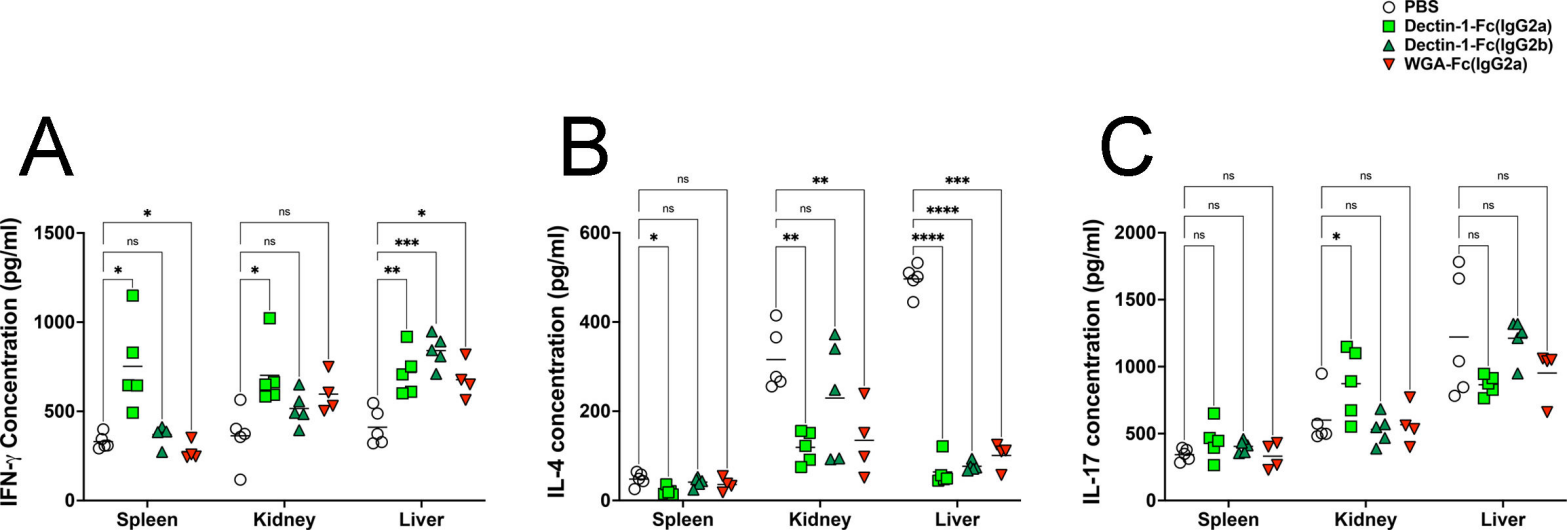
Lectin-Fc(IgG) proteins modulate the cytokine production in a *C. auris* systemic infection model. Cytokine levels were measured in spleen, kidney, and liver organ homogenates at 7 days post-infection by capture ELISA assay. (**A**) IFN-γ levels were significantly elevated in all organs after Dectin-1-Fc(IgG2a) treatment, with similar trends for WGA-Fc(IgG2a) in liver; (**B**) IL-4 levels were suppressed in all organs following Lectin-Fc(IgG) treatment, indicating reduced Th2 polarization; and (**C**) IL-17 levels were selectively increased in the kidneys of Dectin-1-Fc(IgG2a)-treated mice. Symbols: *P < 0.05, **P < 0.01, ***P < 0.001, and ****P < 0.0001.

To assess the immunomodulatory effects of the Lectin-Fc(IgG) treatments during *C. auris* infection, we measured the levels of IFN-γ, IL-4, and IL-17 in the spleen, kidney, and liver at 7 days post-infection. The cytokines IFN-γ, IL-4, and IL-17 were preferentially selected due to their well-established and complementary roles in modulating the immune response, indicating distinct T-helper (Th) immune polarization induced by therapeutic interventions, providing mechanistic insights into protective versus non-protective responses. Dectin-1-Fc(IgG2a) treatment significantly increased the IFN-γ levels in the spleen compared to PBS controls, while WGA-Fc(IgG2a) resulted in an opposite trend (Fig. 7A). Following Dectin-1-Fc(IgG2a) treatment, IFN-γ levels were also elevated in both the kidney and liver (*P* < 0.01). Moreover, all three Lectin-Fc(IgG) treatments increased IFN-γ in the liver.

Regarding IL-4, Dectin-1-Fc(IgG2a) treatment significantly reduced the levels of this cytokine in the spleen, kidney, and liver (Fig. 7B). Similarly, WGA-Fc(IgG2a) treatment decreased the IL-4 levels in the kidney. However, all three Lectin-Fc(IgG) treatments contributed to IL-4 suppression in liver tissues. Finally, IL-17 levels were only augmented in the kidney of Dectin-1-Fc(IgG2a)-treated mice upon infection (Fig. 7C).

## DISCUSSION

*C. auris* has emerged as a major global health threat due to multidrug resistance across all primary antifungal classes, highlighting the urgent need for novel therapeutic and diagnostic strategies (18, 32). Recognition of *C. auris* by mononuclear phagocytes is primarily mediated by Dectin-1, dependent on β-1,3-glucan exposure, with additional contributions from mannose receptor and complement receptor 3 recognizing abundant mannans (33). Despite pathogen-induced cell wall remodeling, β-1,3-glucan remains consistently expressed (34, 35), while echinocandin resistance is often associated with compensatory chitin accumulation (36). These observations underscore the potential of simultaneously targeting β-1,3-glucan and chitin as a rational, synergistic antifungal strategy (37).

In this context, Lectin-Fc(IgG) proteins emerge as a promising dual-function platform with both therapeutic and diagnostic potential for invasive fungal infections due to their broad ability to recognize and bind to polysaccharides present on the cell walls of multiple fungal species (22–24). We exploited the high-affinity binding of the lectins WGA and Dectin-1 to chito-oligomers and β-1,3-glucans, respectively, in combination with the immune-activating function of the IgG Fc effector domain. Their ability to bind a broad range of fungal pathogens suggests potential utility as a preliminary screening tool to differentiate fungal infections from other infectious etiologies. However, further studies are required to assess specificity, distinguish colonization from active infection, and clarify their integration alongside species-specific diagnostic methods for guiding targeted treatment decisions. By accelerating fungal detection and providing therapeutic benefits, Lectin-Fc(IgG) proteins have the potential to reduce the risk of rapid fungal dissemination and improve patient outcomes.

Our study demonstrates that engineered Lectin-Fc(IgG)s exert protective effects against *C. auris*. These findings support the potential of immunotherapeutic approaches that utilize innate immune recognition to boost host defense and mitigate fungal pathogenesis. Lectin-Fc(IgG)s, particularly Dectin-1-Fc(IgG2b), exhibit a robust and concentration-dependent binding to β-1,3-glucan in *C. auris*, as also seen for *C. albicans*. In contrast, WGA-Fc(IgG2a) displayed low binding affinity to *C. auris* compared to *C. albicans* (22). Dectin-1-Fc(IgG)s and WGA-Fc(IgG2a) binding to *C. auris* predominantly concentrated in the budding regions along the intercellular septa, reflecting increased exposure of chitin and β-glucan in these regions. However, WGA-Fc(IgG2a) labeled both yeasts in the budding regions, with lower intensity in *C. auris* than *C. albicans*. Our observations are in accordance with previous results highlighting the unique cell wall architecture of *C. auris* in comparison to other *Candida* spp. (33), characterized by the reduced chitin level, which reflects in higher adaptive plasticity and flexibility of the cell wall under osmotic and other environmental stressors (38). However, *C. auris* may form multicellular aggregates—a phenotype that distinguishes numerous clinical isolates from the typical single-celled yeast form—can be induced under stressful *in vitro* or *in vivo* conditions (39, 40). Such activated regulatory mechanisms involve cell wall remodeling and morphological changes that promote an aggregative phenotype, representing a virulence-associated adaptive response, featuring higher chitin exposure (39, 40), also suggesting the potential use of these Lectin-Fc(IgG)s in *in vivo* models. 

Lectin-Fc(IgG) fusion proteins demonstrated direct antifungal activity against *C. auris in vitro,* exerting predominantly fungistatic effects. Remarkably, both subclasses of Dectin-1-Fc(IgG) significantly restricted fungal proliferation and reduced biofilm formation in a clear dose-dependent manner. By contrast, WGA-Fc(IgG) exhibited only marginal and discrete effects on growth inhibition, likely reflecting the limited exposure of chitin within the fungal cell wall, as previously described. Biofilms from *C. auris* are robust structures linked to fungal persistence in the environment and healthcare settings, as well as its multidrug resistance (41, 42). The evident fact that Lectin-Fc(IgGs) were able to reduce biofilm formation as early as 24 h and persisted through 48 h reinforces their relevance against fungal drug-resistant phenotypes, such as *C. auris*.

The Lectin-Fc(IgG)s developed by our group offer distinct advantages over native lectins (which rely only on recognition and complement deposition) and classical antifungals, which would lack potential immunological synergism (43). One of their key advantages is the presence of the Fc portion, which facilitates immune interactions and antibody-dependent cellular toxicity (ADCC) through Fc gamma receptors, potentially improving their half-life *in vivo* (43). Given their opsonic properties, Lectin-Fc(IgG) proteins may also contribute to complement activation, in addition to activating immune cells, thereby enhancing immune defense against fungal infections (33). By engaging multiple immune receptors, Lectin-Fc(IgG) proteins amplify immune system activation, surpassing the limitations of conventional antifungal treatments.

Previous studies have demonstrated the immunomodulatory effects of the antibody-like Lectin-Fc(IgG)s during fungal infections. These include the enhancement of alveolar macrophage antifungal activity through opsonization, leading to increased phagocytosis of *A. fumigatus*, *C. albicans*, *H. capsulatum*, and *C. neoformans* (22–24), the promotion of complement activation via elevated C3 deposition on the fungal surface (23, 24), and the enhancement of dendritic cell antigen presentation, resulting in increased secretion of IL-6, IL-10, and TNF-α in response to *C. albicans* (44). Consistent with these findings, our results show that Lectin-Fc(IgG) proteins enhance macrophage interactions with *C. auris* yeast cells, leading to enhanced fungal killing *in vitro*.

Our *in vivo C. auris* infection model further supports the therapeutic antifungal potential of Lectin-Fc(IgG) proteins. Mice treated with these proteins showed significantly improved survival rates up to 30 days post-systemic infection with *C. auris*, mitigating clinical signs, including weight loss, along with a reduced fungal burden in all the organs evaluated. During systemic infection, *C. auris* rapidly develops an adaptive multicellular aggregative morphology. These aggregates accumulate in multiple organs, including the brain, liver, and spleen, and are associated with the regulation of cell wall integrity, cytokinesis, and cytoskeletal properties, factors that may alter cell wall composition and enhance fungal virulence (40). Importantly, such cell wall reorganization may also increase glucan and chitin exposure, thereby facilitating recognition by Lectin-Fc(IgG) proteins and amplifying the host immune response (45).

Although WGA-Fc(IgG2a) exhibited lower binding affinity and more modest antifungal and antibiofilm effects *in vitro* compared with the β-1,3-glucan-targeting Dectin-1-Fc(IgG) counterparts, it conferred the strongest protection *in vivo*, producing a pronounced 2 log reduction in hepatic fungal burden. While the *in vitro* assays capture binding, direct fungistatic effects, and opsonin properties, the *in vivo* context also allows the engagement of Fc-dependent effector mechanisms, including complement activation and ADCC, which may compensate for lower glycan-binding affinity. Additionally, fungal cell wall remodeling under host-induced stress may enhance the accessibility of chitin-rich regions targeted by WGA-Fc(IgG2a), particularly in infected tissues (33, 40, 46). Together, these mechanisms explain the unexpectedly robust *in vivo* phenotype of WGA-Fc(IgG2a), aligning with previous findings from *in vivo* models of candidiasis by *C. albicans*, as well as in histoplasmosis, cryptococcosis, and aspergillosis (22–24).

Cytokine provides additional insights into the immunomodulatory effects of ectin-Fc(IgG) proteins by shifting the profile toward a pro-inflammatory antifungal response. We selected IFN-γ, IL-4, and IL-17 for cytokine profiling based on their pivotal roles in orchestrating antifungal immunity and their ability to reflect the polarization of host immune responses. IFN-γ is a hallmark Th1 cytokine essential for macrophage activation, fungal killing, and protective cell-mediated immunity against invasive *Candida* infections. IL-4, in contrast, is a representative Th2 cytokine associated with non-protective or detrimental responses in fungal infections, often antagonizing IFN-γ activity. IL-17, a signature Th17 cytokine, is critical for neutrophil recruitment and mucosal defense and has been implicated in protection against *Candida* spp., including *C. auris*. While other pro-inflammatory cytokines such as TNF-α, IL-1β, and IL-6 are also relevant, they are primarily upstream or general mediators of inflammation and not as directly indicative of T-helper subset polarization. All three Lectin-Fc(IgG) treatments significantly increased IFN-γ levels in the liver, a critical cytokine for macrophage activation and fungal clearance, suggesting a common immunostimulatory effect in this organ. Additionally, all treatments suppressed IL-4 production, indicating a shift away from Th2-mediated non-protective immune responses. Notably, Dectin-1-Fc(IgG2a) uniquely induced IL-17 levels in the kidney, suggesting localized Th17 activation, which has been shown to enhance mucosal and tissue-level immunity, leading to fungal clearance (47, 48). These results suggest that fungal clearance is likely mediated by a combination of direct antifungal effects of Lectin-Fc(IgG)s and immune activation, including enhanced macrophage function, as inferred from cytokine profiles.

Altogether, our findings establish Lectin-Fc(IgG)s as modulating immunobiologicals with multiple antifungal activities against the multidrug-resistant *C. auris*. Their ability to selectively target vital fungal cell wall components, modulate the immune response, and enhance host defense reinforces their potential as a novel strategy for combating invasive fungal infections.

From a translational standpoint, Lectin-Fc(IgG)s hold promise for rapid deployment in immunocompromised patients, who often cannot mount effective vaccine responses. Their use as passive immunotherapeutics, either alone or in combination with existing antifungal drugs, may offer synergistic protection while minimizing the emergence of resistance. Future evaluations against *C. auris* should include strains from distinct clades, pharmacokinetic optimization and combinations with antifungals, additional pharmacodynamics evaluation, large-scale production, and toxicological safety. Furthermore, their potential as diagnostic tools, particularly for early detection of invasive mycoses where culture-based methods fall short, should also be validated. Ultimately, Lectin-Fc(IgG) proteins represent a versatile and promising strategy to complement the portfolio of developing drugs to address the urgent global threat posed by *C. auris*.
